# Hypodermic Administration of Medical Agents

**Published:** 1867-04

**Authors:** 


### HYPODERMIC ADMINISTRATION OF MEDICAL AGENTS.

We are in receipt of a valuable contribution for the Journal, on this subject, from an intelligent and reliable physician of Georgia. We regret that it did not reach us in time to appear in the present issue. It will have a place in the May number of our Journal.

This mode of administering remedies, though for sometime practiced, and made the subject of especial study and experiment by some, has not been so impressed upon the profession as to make it a matter of general interest. Very few have given the subject much thought; and a still smaller number of physicians have supplied themselves with an instrument for the purpose. The forthcoming article, alluded to above, will give the desired information and directions to those who have not made themselves familiar with the subject.

The following extracts from a lecture on this subject, by Edward Warren, M. D., late Surgeon General of North Carolina, delivered before the Baltimore Medical Association, we commend to our readers:—

"In the early stages of *pneumonia*, *pleurisy*, *bronchitis*, *peritonitis*, *enteritis*, *phlebitis*, and inflammations generally, when congestion exists, and exudation has not occurred—when the pathological condition consists in a local irritation, with hyperæmia resulting from perverted nervous action—the subcutaneous injection of morphia plays an important role in scientific therapy.

"This practice is particularly indicated when the excitement of the nervous system is altogether disproportional to the exaggeration of vascular action, as is indicated by violent pain, increased sensibility to local impressions, and disorders generally of the sensory and motor functions.

"Again, in inflammations of such organs as are largely supplied with ganglionic nerves, and in the treatment of which the nervous system requires an unusual share of attention, this mode of employing morphia may be used to advantage.

"Without discussing the essential nature of inflammation, I would direct your attention briefly to the consideration of the part played by the nervous system in the development of its characteristic phenomena.

"1. Heat, local and general, is one of the earliest and most persistent of the symptoms or signs of the inflammatory process.

"Since the experiment of Sir Benjamin Brodie, in 1811, it has been recognized as a physiological axiom, that the nervous system, though not a generator of heat, *per se*, exercises a controlling influence over those local processes of nutrition and the metamorphosis of tissues, by which the work of calorification is effected in the human organism.

"2. Alterations in the tension and velosity of the blood stream speedily manifest themselves in this regard.

"BERNARD has demonstrated that "certain parts of the nervous system preside over and regulate the general and local circulations," and the fact is universally admitted at the present day.

"Though the heart possesses an inherent contractility, it is also liberally furnished with nerves, both from the cerebro-spinal and the ganglionic systems, which separately and conjointly exercise a potential influence over it.

"The arteries are similarly supplied with nerves, as has been shown by the investigations of BERNARD, BUDGE, SEQUARD, SCHIFF, and others; while VALENTINE has proven by experimental research, that the veins and larger lymphatic trunks are similarly endowed.

"Even the power of contraction which resides in the capillaries, is influenced by the condition of the ganglionic nerves distributed to them, as is evinced in actions which are essentially nervous, such as the flushing of the countenance from mental emotions, etc., etc.

"3. Disturbances in the secerning organs are the ordinary concomitants of inflammation.

"The influence of the nervous system on the secerning organs is indisputable. BERNARD has learned to stimulate or repress them at will, by exciting particular parts of the nervous system. 'Glands are not filters,' says that distinguished physiologist, 'but organs producing chemical substances, under *influence of the nervous system.*

"4. Certain phenomena, essentially nervous in their character, also present themselves in this connection, such as wakefulness, insomnia, pain, augmented sensibility to impressions, delirium, trembling, etc., etc.

"As the normal phenomena alluded to under these various heads are known to occur under the superintending guidance and direction of the nervous system, it is but rational to consider all morbid actions in these connections as being influenced in their manifestations by aberrated nervous action.

"Thus augmentations of temperature result from an increased activity of the causes which operate in the production of physiological or healthy calorification, and indicate some positive change—some decided disturbance—in the nervous system. As the essential nature of the febrile state has been recognized, since the days of GALEN, to consist in a *calor prœter naturem*, it follows that *fever*, which is the most universal and important of all the symptoms of inflammation, is in itself a phenomenon of perverted or disturbed nervous action.

"In the same way it can be shown that all morbid changes, alluded to as occurring alike in the circulatory system, in the secerning organs, and in the nerves themselves, are similarly produced, and possess the same pathological significance.

"Whatever may be the nature of this *disturbance* in the nervous system, the effect of the hypodermic administration of morphia is to calm it. It seems to coerce the whole organism—centers, filaments, and all—into a state of profound quiescence. It establishes throughout the entire system one uniform standard or condition of vitality, to which every cell, tissue, organ, and function is compelled to conform, and through the instrumentality of which the morbid processes are suspended or paralyzed.

"I have repeatedly cured *intermittents* of the most persistent character by injecting morphia subcutaneously a short period in advance of the expected paroxysm or after its development, and repeating the operation on the seventh day of several succeeding weeks. Morphia is much less irritating to the tissues than quinia, while its effects in this connection are not the less satisfactory.

In the exacerbation of *remittents*, when attended with violent retching, severe muscular pain, great restlessness, and even intense headache, I have often witnessed an almost instantaneous abatement of all the symptoms, together with the development of a copious perspiration after the introduction under the skin of a full dose of morphia.

"In *typhoid fever*, when the patient is inordinately "nervous," restless, morbidly vigilant, tremulous, peculiarly sensitive to morbid impressions, etc., this remedy answers well.

"You have doubtless attended cases of this disease, in which many of the symptoms of cerebritis presented themselves, when your judgments convinced you in advance, and your subsequent *post-mortem* examinations demonstrated that the morbid lesion existed exclusively in the intestines. The delirium thus developed through the instrumentality of nervous reflex action, furnishes no argument against the use of opium, but rather an indication for its employment.

"In a case of *intussusception*, occurring in the practice of my father, Dr. Wm. C. Warren, after stercoraceous vomiting had occurred, and innumerable remedies having been tried in vain, an injection of morphia under the skin afforded almost instant relief. * * * * *

"The hypodermic employment of morphia possesses equal curative properties in many affections which pertain exclusively to the domain of *surgery*; and I must ask your indulgence, while briefly recapitulating my experience in that regard.

"In several cases of *strangulated inguinal tumor*, when ordinary remedies had failed to induce a sufficient relaxation to render a return of the intestine by taxis possible, I have promptly overcome the spasm, by injecting morphia immediately *over the point of* constriction. These results were due to the direct sedative or narcotic action of the drug, and to nothing else.

"Last summer I was consulted by a young man who was suffering witn *orchitis* in its primitive stage. There was already considerable swelling, together with great tenderness, pain, and redness of the part.

"Half a grain of the sulphate of morphia in solution, was injected directly over the track of the cord as it passes from the inguinal canal, and the patient placed in his bed. On the succeeding morning, I found that he had slept profoundly during the entire night, and that not a trace of inflammation remained.

"In the same way, and with equal success, I have treated traunatic *erysipelas*—which is frequently nothing more than an acute cutitis—a tendency to *gangrene* from excessive local inflammation, and *phlebitis* of a very decided character.

"During the war, I was summoned hurriedly on one occasion, to a man who had received a deep *wound of the right lung*, from a bowie knife in the hands of a drunken companion. The usual symptoms of this accident presented themselves, and the patient was in a very critical condition. Turning him upon the opposite side, I injected a grain of morphia in solution under the skin, and, so soon as sleep was induced and the hæmorrhage restrained, closed the wound hermetically. There was no subsequent bleeding, the respiratory movements were sufficiently restrained, but little cough was developed, no inflammatory symptoms appeared, and under the repeated use of the remedy originally employed, the case rapidly progressed to a favorable conclusion.

"Within a few days, I have attended a patient who was suffering *spasmodic stricture*, which had been greatly increased by repeated attempts to introduce a catheter. His bladder was greatly distended, and the pain excessive. An injection of half a grain morphia into the arm, immediately relaxed the spasm, and restored the young man to health.

"From these instances of the curative action incident to this mode of medication, as well as from many others to which I might refer, if time and space permitted, it is plain, that for the relief of pain, the relaxation of spasm, the abatement of irritation, the induction of sleep, the control of the secerning organs, and the restraint of certain forms of inflammation in their primitive stage, this remedy deserves to stand *primus inter pares* in the estimation of an enlightened and progressive profession."

